# Supplementary material for: Trypstatin as a Novel TMPRSS2 Inhibitor with Broad‐Spectrum Efficacy against Corona and Influenza Viruses
Source: Adv Sci (Weinh). 2025 May 14;12(25):2506430. doi: 10.1002/advs.202506430 (PMC12224939; doi:10.1002/advs.202506430)
Supplement: Supplementary file 1 — Supporting Information [file ADVS-12-2506430-s001.docx]

**Supporting Information**

**Trypstatin as a Novel TMPRSS2 Inhibitor with Broad-Spectrum Efficacy Against Corona and Influenza Viruses**

Jan Lawrenz^1^, Lukas Wettstein^1^, Armando Rodríguez Alfonso^2,3^, Rayhane Nchioua^1^, Pascal von Maltitz^1^, Dan P.J. Albers^1^, Fabian Zech^1^, Julie Vandeput^4^, Lieve Naesens^4^, Giorgio Fois^5^, Veronika Neubauer^5^, Nico Preising^2^, Emilia Schmierer^1^, Yasser Almeida-Hernandez^6^, Moritz Petersen^7^, Ludger Ständker^2^, Sebastian Wiese^3^, Peter Braubach^8^, Manfred Frick^5^, Eberhard Barth^9^, Daniel Sauter^7^, Frank Kirchhoff^1^, Elsa Sanchez-Garcia^6^, Annelies Stevaert^4^ and Jan Münch^1,2#^

^1^Institute of Molecular Virology, Ulm University Medical Center, 89081 Ulm, Germany

^2^Core Facility Functional Peptidomics, Ulm University Medical Center, 89081 Ulm, Germany

^3^Core Unit Mass Spectrometry and Proteomics, Ulm University Medical Center, 89081 Ulm, Germany

^4^Rega Institute for Medical Research, Department of Microbiology, Immunology and Transplantation, KU Leuven, 3000 Leuven, Belgium

^5^Institute of General Physiology, Ulm University, 89081 Ulm, Germany

^6^Computational Bioengineering, Biochemical and Chemical Engineering, TU Dortmund University, 44227 Dortmund, Germany

^7^Institute for Medical Virology and Epidemiology of Viral Diseases, University Hospital Tübingen, 72076 Tübingen, Germany

^8^Institute of Pathology, MH Hannover, 30625 Hannover, Germany

^9^Anesthesiology and Intensive Medicine Clinic, Ulm University Medical Center, 89081 Ulm, Germany

^#^Corresponding author:

Jan Münch, Institute of Molecular Virology, Meyerhofstrasse 1, 89081 Ulm, Germany, phone: +49 731 500 65154, E-mail: [jan.muench@uni-ulm.de](mailto:jan.muench@uni-ulm.de)

**
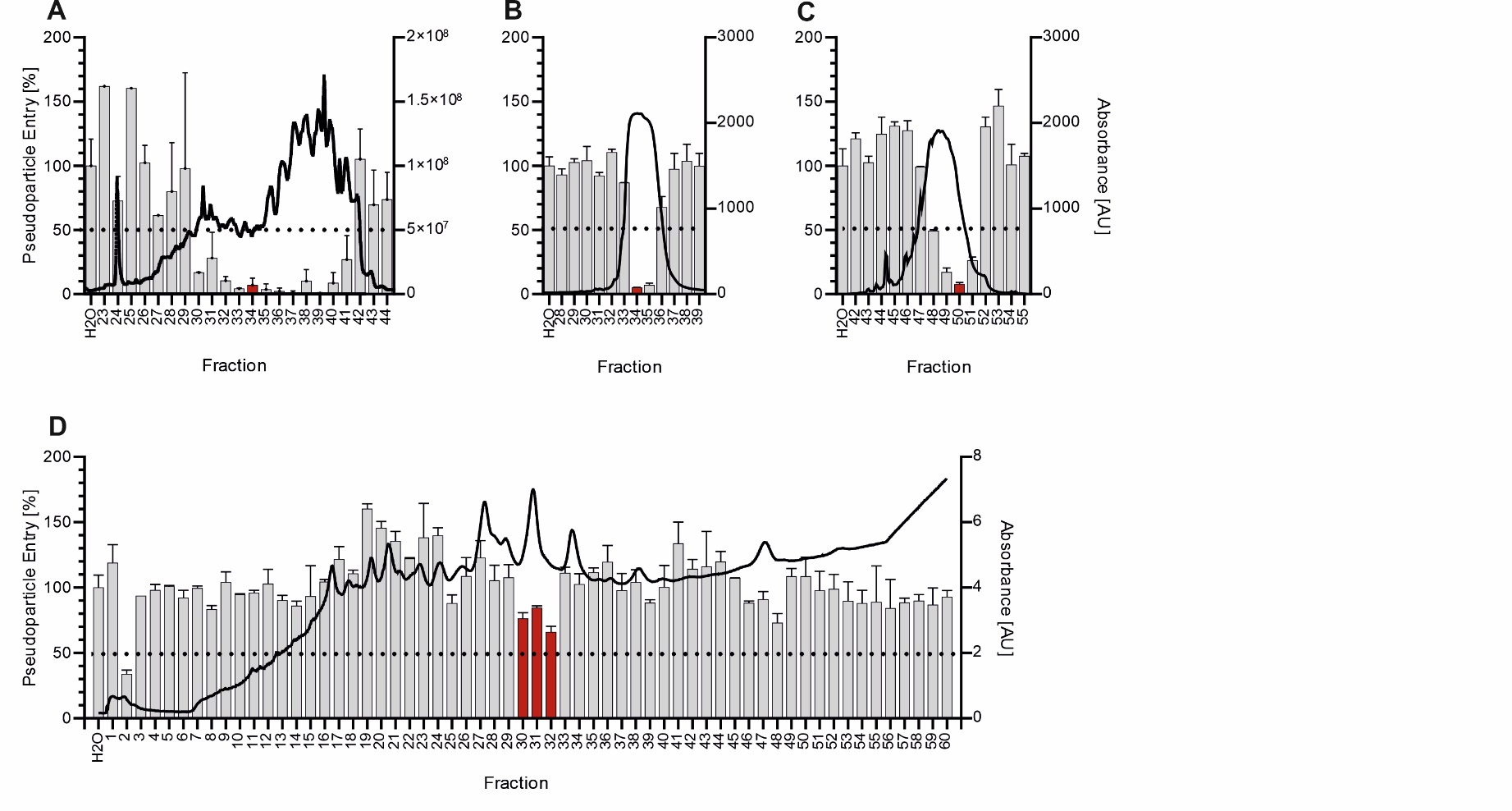
Figure S1: High-throughput screening for endogenous inhibitors of SARS-CoV-2 entry.** **(A-D)** Caco-2 cells were pre-treated with peptide/protein containing fractions of subpurified hemofiltrate library fractions before transduction with luciferase-encoding lentiviral pseudoparticles harboring the SARS-CoV-2 Hu-1 spike. Transduction rates were assessed 48 h later by measuring luciferase activity in cell lysates. Columns represent pseudoparticle entry and black line absorbance at 280 nm of the corresponding fraction. Fractions marked in red (A-C) were subpurified before final fractions **(D)** were analyzed by LCMS/MS. Shown are mean values of one experiment performed in triplicates ± SEM.


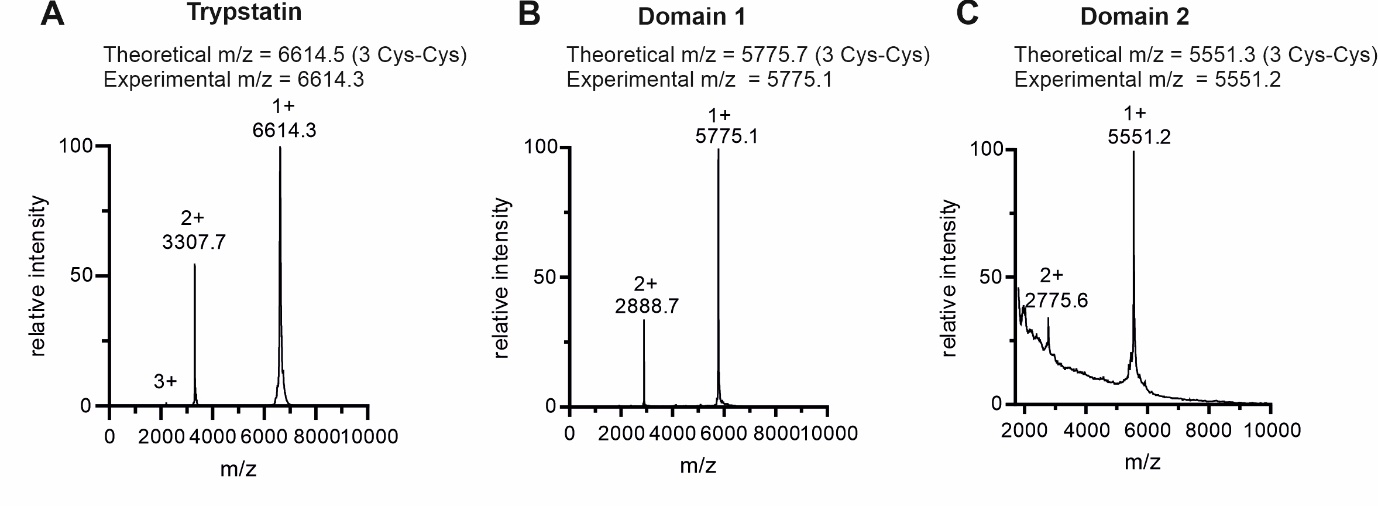
**Figure S2: MALDI-TOF-spectra of synthetically produced and refolded kunitz domains.**  **(A)** Trypstatin (Protein AMBP, P02760: residues 284-344), **(B)** BPTI/Kunitz inhibitor 1 domain (Protein AMBP, P02760: residues 231-281), (C) BPTI/Kunitz inhibitor 2 domain (Protein AMBP, P02760: residues 287-337). In all cases, the experimental average masses indicated the formation of three disulfide bridges.

**
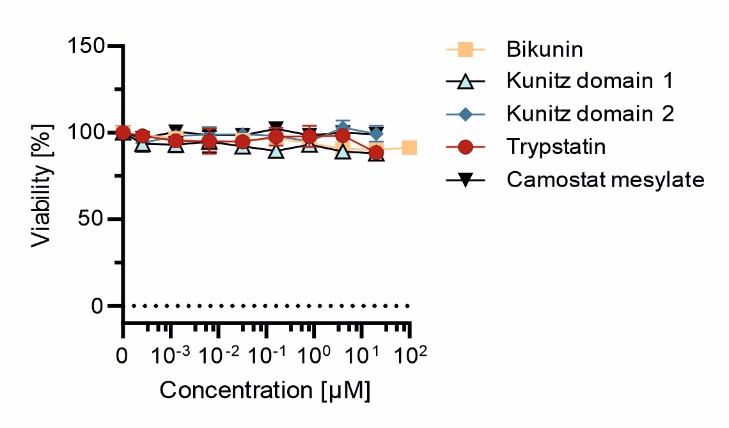
**

**Figure S3: Trypstatin does not influence cell viability.** Caco-2 cells were treated with serial dilutions of compounds for 48 h before cell viability was assessed using the Promega CellTiter-Glo® Cell Viability Assay. Shown are mean values of three independent experiments performed in triplicates ± SEM.


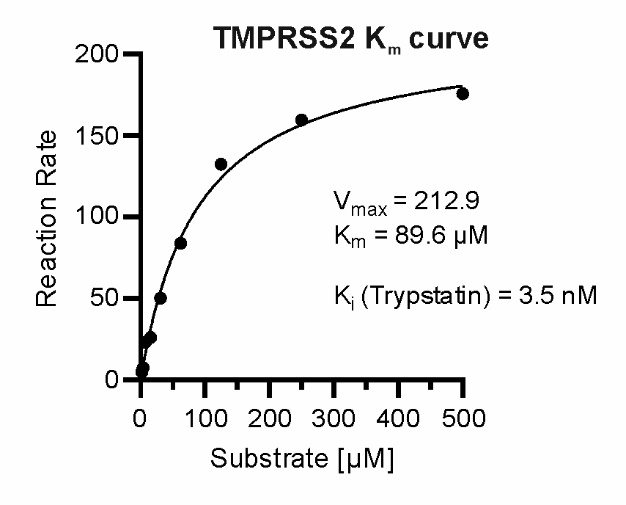


**Figure S4: Michaelis constant (K_m_) of the fluorogenic reporter substrate Boc-Gln-Ala-Arg-AMC for TMPRSS2.** Boc-QAR-AMC was serially diluted in assay buffer with a final TMPRSS2 concentration of 20 nM, and initial velocity was measured in 50-s intervals for 15 min with excitation of 360 nm and emission of 460 nm on a Synergy plate reader. K_m_ and V_max_ were calculated from Michaelis−Menten plots using GraphPad Prism. Shown are mean values of one experiment performed in quadruplicates.

**
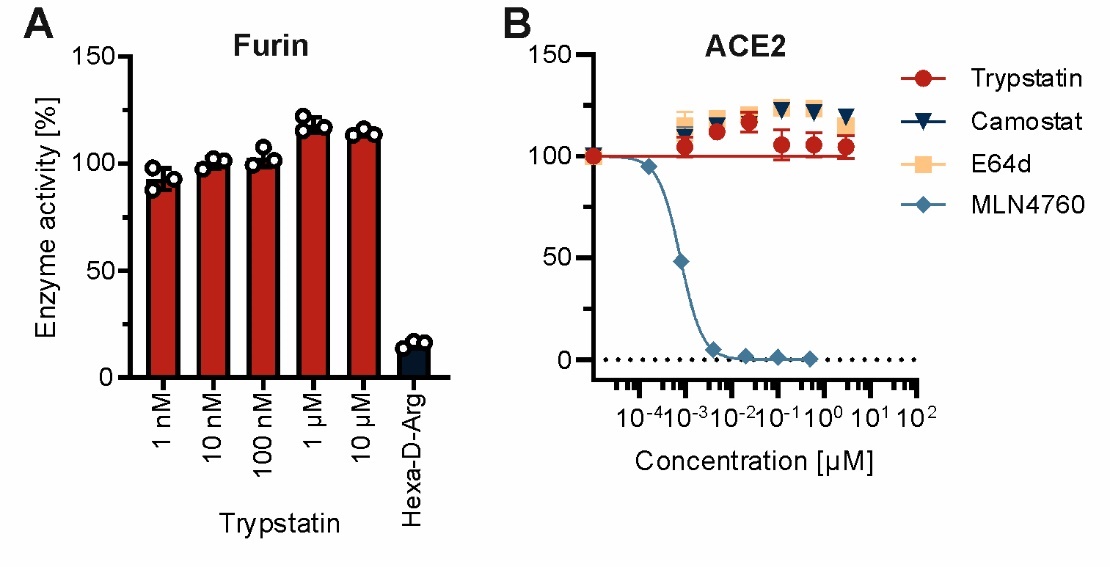
**

**Figure S5: Trypstatin does not inhibit furin or ACE2.** Compounds were mixed with recombinant furin or ACE2 before addition of a fluorogenic reporter substrate. Fluorescence intensity was measured at an excitation wavelength of 355 nm and emission wavelength of 460 nm. Shown are mean values of three independent experiments performed in duplicates ± SEM.

**
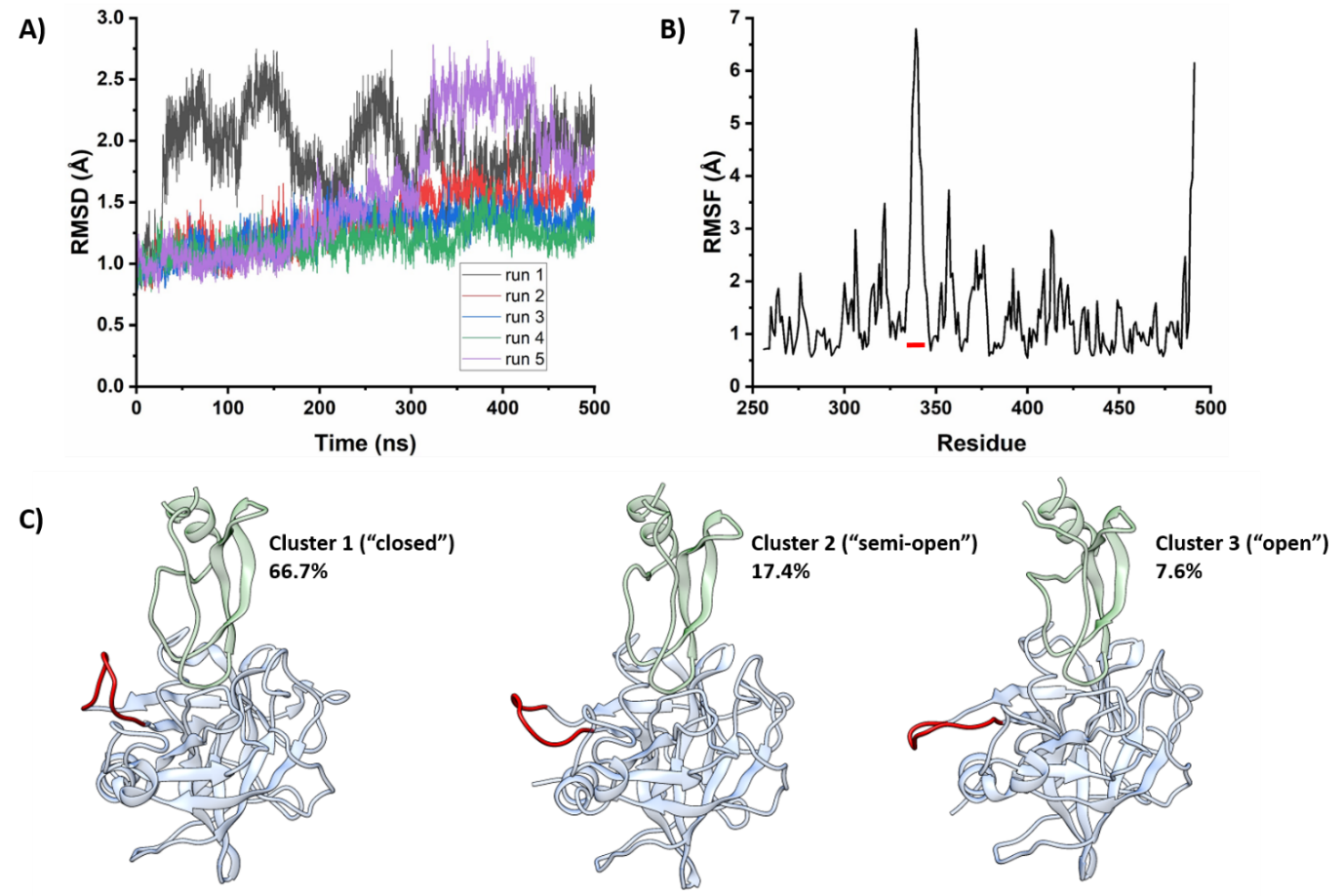
Figure S6: The simulations showed the dynamic behaviour of the backbone atoms of TMPRSS2. (A)** RMSD evolution of the backbone atoms of TMPRSS2. (**B)** The RMSF analysis shows the high flexibility of the loop (highlighted by the red line) encompassing residues 336-345 of TMPRSS2. (**C)** Representative structures from the clustering of the TMPRSS2’s 336-345 loop (red). The clustering analysis of this loop showed three clusters of structures, which can be classified according to the relative orientation with respect to Trypstatin. The percent refers to the cluster size of the sampled population during 1.5 µs. The most populated cluster of structures (66.7%, the loop is highlighted in red), displays a “closed” conformation of the loop (“near” Trypstatin), followed by a cluster with the loop positioned on an intermediate orientation with respect to Trypstatin (semi-open, 17.4%), and other cluster of structures with the loop adopting an “open” conformation, further away from Trypstatin (7.6%). Although “open” complexes represented only 7.6% of the total frames, they were prevalent in replicas 1 and 5 of the simulations. RMSD = root mean sqare deviation, RMSF = root mean square fluctuation.

**
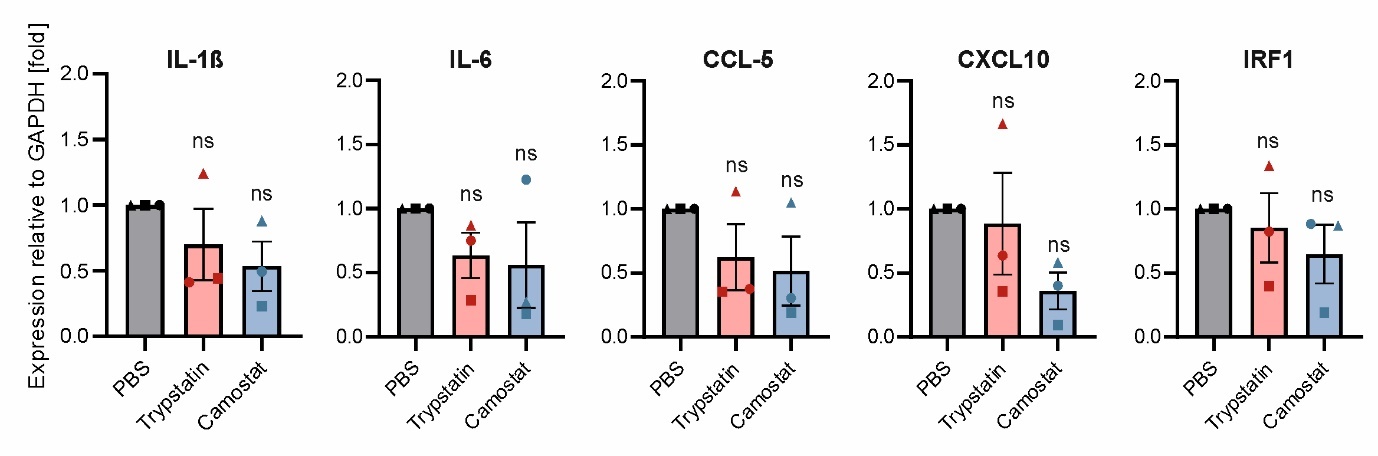
**

**Figure S7: Trypstatin does not have a significant anti-inflammatory effect in SARS-CoV-2 infected HAECs.** Human airway epithelial cells (HAECs) were infected with SARS-CoV-2 Omicron BA.5 while being exposed to the respective inhibor before 2 days post infection cells were lysed and expression of respective genes were analyzed by RT-qPCR. Shown are mean values of duplicates per donor ± SEM. Each symbol (triangle, circle, square) represents one donor. One-way-ANOVA was applied to test for significance.

**
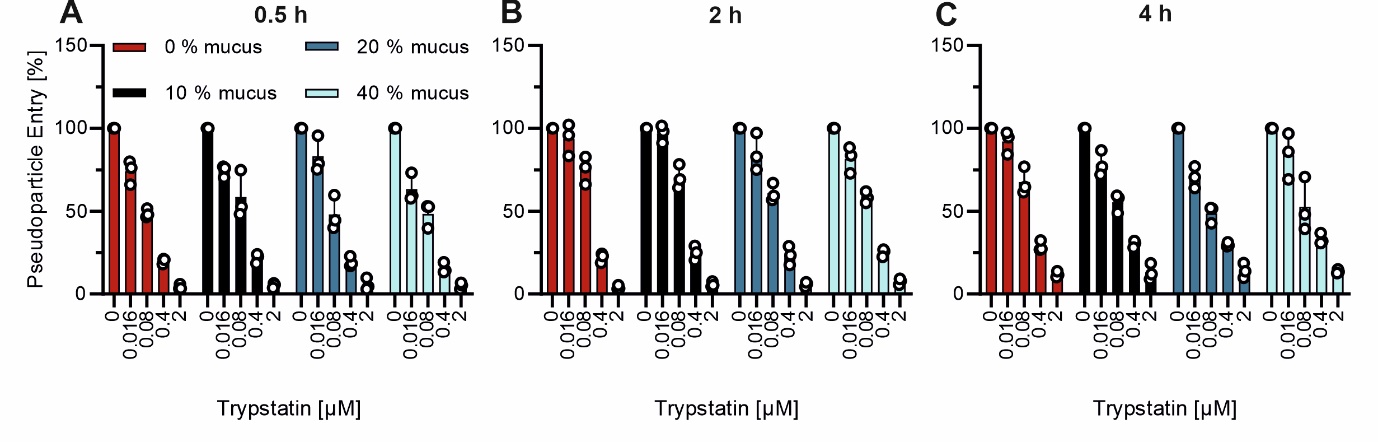
**

**Figure S8: Trypstatin remains active in the presence of human airway mucus.** Serial dilutions of Trypstatin were mixed to the indicated concentrations with mucus from HAEC cultures at 37°C for 30 min (A), 2 h (B) or 4 h (C) before addition to Caco-2 cells and subsequent transduction with luciferase encoding lentiviral pseudoparticles harboring the SARS-CoV-2 Hu-1 spike. Transduction rates were assessed 48 h later by measuring luciferase activity in cell lysates. Shown are mean values of experiment performed in triplicates ± SEM.

**
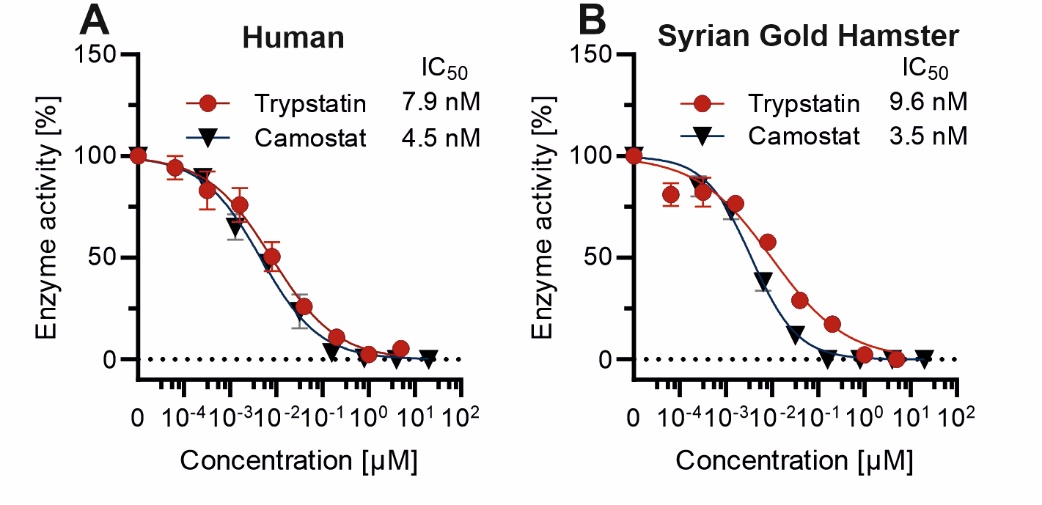
**

**Figure S9: Trypstatin blocks hamster and human TMPRSS2 activity with similar efficacies.** HEK293T-cells were transfected with human or hamster TMPRSS2 expression plasmid or mock control, respectively, before treatment with serial dilutions of inhibitors and the addition of a fluorogenic reporter substrate. Values were corrected for the signal of mock-transfected HEK293T-cells. Shown are mean values of one experiment performed in triplicates ± SEM.


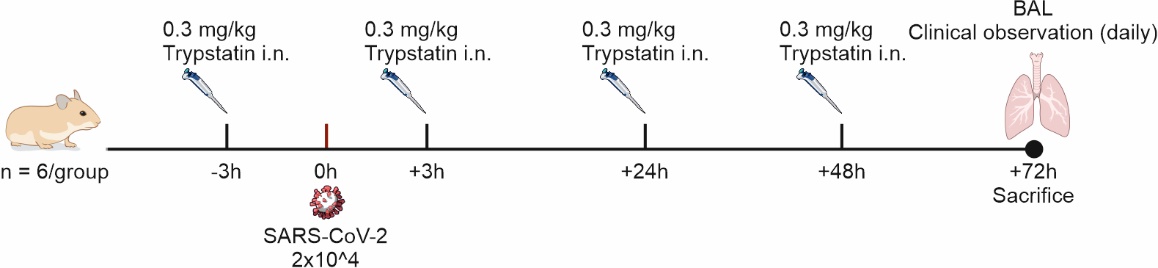


**Figure S10: Treatment schedule of *in vivo* study.** 12 syrian gold hamsters were randomized into groups of 6. Groups were either treated with 0.3 mg/ml Trypstatin or PBS at the indicated time points. Hamsters were inoculated with the SARS-CoV-2 delta variant. 72h post-infection, hamsters were sacrified and bronchoalveolar lavage was taken. Clinical symptoms were recorded on a daily basis.

|  | **SARS-CoV-2 Hu-1 pp** | **SARS-CoV-2 B.1** | **SARS-CoV-2 BA.1** | **SARS-CoV-1 pp** | **MERS-CoV pp** | **hCoV-NL63** | **IAV H1N1** | **IAV H3N2** | **IBV** |
| --- | --- | --- | --- | --- | --- | --- | --- | --- | --- |
| **Trypstatin** | 133.2 ±11 | 26.4 ±4 | 124.0 ±11 | 67.9 ±13 | 78.1 ±13 | 195.2 ±42 | 292.5 ±79 | 144.0 ±41 | 570.3 ±168 |
| **Camostat mesylate** | 93.8 ±6 | 4.1 ±2 | 68.1 ±5 | 71.1  ±8 | 49.9 ±19 | 109.6 ±24 | 219.8 ±49 | 50.2 ±16 | 125.1 ±33 |
| **Nafamostat mesylate** | 14.4 ±1 | n.d. | n.d. | n.d. | n.d. | n.d. | n.d. | n.d. | n.d. |
| **Aprotinin** | 427.4 ±30 | n.d. | n.d. | n.d. | n.d. | n.d. | 2254 ±886 | 1456 ±693 | 2789 ±871 |
| **Bikunin** | 6100 ±1200 | n.d. | n.d. | n.d. | n.d. | n.d. | n.d. | n.d. | n.d. |
| **Bikunin KD 1** | - | n.d. | n.d. | n.d. | n.d. | n.d. | n.d. | n.d. | n.d. |
| **Bikunin KD 2** | 170.2 ±22 | n.d. | n.d. | n.d. | n.d. | n.d. | n.d. | n.d. | n.d. |
| **Antitrypsin** | 27400 ±4100 | n.d. | n.d. | n.d. | n.d. | n.d. | n.d. | n.d. | n.d. |
| **Antithrombin** | 1500 ±180 | n.d. | n.d. | n.d. | n.d. | n.d. | n.d. | n.d. | n.d. |
| **MM3122** | 31.2 ±3 | n.d. | n.d. | n.d. | n.d. | n.d. | n.d. | n.d. | n.d. |
| **Nirmatrelvir** | n.d. | n.d. | n.d. | n.d. | n.d. | 1260 ±64 | n.d. | n.d. | n.d. |
| **Baloxavir acid** | n.d. | n.d. | n.d. | n.d. | n.d. | n.d. | 1.6 ±1 | 2.1 ±52 | 10.3 ±2 |

**Table S1: Summary of all anti-viral IC_50_ values in nanomolar (±SEM) presented in the manuscript.** pp = pseudoparticle; n.d. = not determined/detectable.
